# Posterior to anterior distal locking of humeral intramedullary nails

**DOI:** 10.1308/003588412X13373405387096d

**Published:** 2012-05

**Authors:** WJ White, RMA Hawken, NC Giles

**Affiliations:** Royal Devon and Exeter NHS Foundation Trust,UK

## BACKGROUND

Distal locking of long intramedullary humeral nails is achieved using a freehand technique. Both anterior-posterior and lateral-medial locking techniques have been linked to neurovascular complications including radial and lateral cutaneous nerve injuries.^1–5^ We describe a technique that is simple to adopt and avoids these complications.

## TECHNIQUE

**Figure 1:**
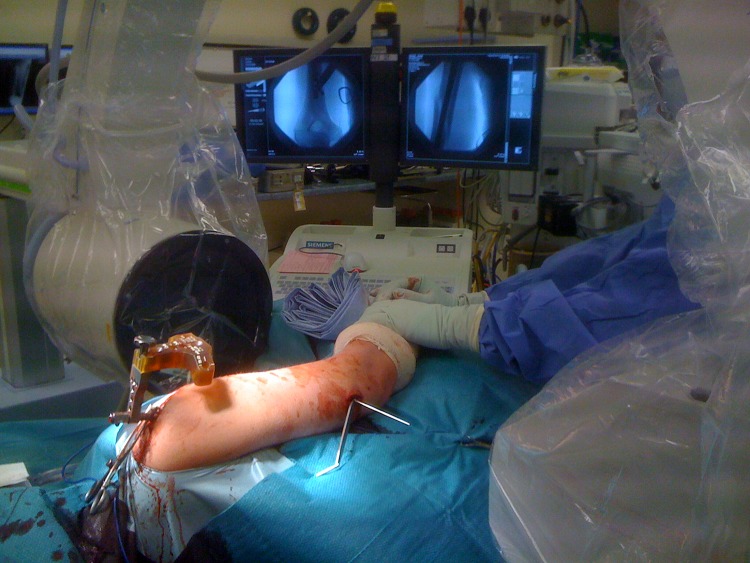
Operative setup for posterior to anterior distal locking

Humeral nailing is performed with the patient in the lateral decubitus position with the image intensifier at 90º to the patient (Fig 1). During freehand distal locking, the patient’s arm rests on his or her body and the forearm is rested on the anterior pelvic support on sterile padding. By changing the amount of padding, arm rotation can be adjusted to give a perfect view of the distal locking hole without requiring an assistant. In this position, we then perform distal locking in a posterior-anterior direction.

## DISCUSSION

At the level of humeral nail distal locking, there are no major neurovascular structures at risk in the posterior aspect of the arm.^4^ Only by drilling through the anterior cortex and the brachialis, potentially damaging the brachial artery and median nerve, could a neurovascular structure be injured using this method. At this level, the posterior humerus has a flatter surface than laterally or anteriorly, reducing the tendency of the tip of the drill to slide and cause iatrogenic injury. This method has technical and safety advantages over other techniques.
